# Development and Implementation of a Professional Practices Evaluation during Radiopharmaceuticals Administration

**DOI:** 10.3390/healthcare10112247

**Published:** 2022-11-10

**Authors:** Charlotte Donzé, Léa Rubira, Lore Santoro, Pierre Olivier Kotzki, Emmanuel Deshayes, Cyril Fersing

**Affiliations:** 1 Nuclear Medicine Department, Institut Régional du Cancer de Montpellier (ICM), University Montpellier, 34298 Montpellier, France; 2 Institut de Recherche en Cancérologie de Montpellier (IRCM), INSERM U1194, Institut Régional du Cancer de Montpellier (ICM), University Montpellier, 34298 Montpellier, France; 3 IBMM, University Montpellier, CNRS, ENSCM, 34293 Montpellier, France

**Keywords:** Nuclear Medicine, radiopharmaceuticals, drug administration, professional practices evaluation, cancer patients management, quality of healthcare

## Abstract

Securing both the patient and radiopharmaceuticals (RPs) circuit is an essential concern in nuclear medicine (NM). These circuits converge at the RP administration phase, a key step in patient management in NM. In a continuous quality improvement approach, we developed and implemented an evaluation of professional practices (EPPs) methodology focused on RPs injection to identify and correct deviations from good practices. The nuclear medicine technologists (NMTs) of a single center were evaluated. A specific audit grid was designed for this purpose, covering 4 main themes. Following the audit campaign, an improvement action plan was set up to address the non-conformities observed. Nine NMTs were audited on 4 RPs injections each. The mean total score was 93.36% with, on average, 7.01% and 3.00% of unmet and partially met criteria, respectively. In view of the non-compliance rates of hygiene and radiation protection items, theoretical reviews of these themes were included in the improvement action plan. As a part of the quality assurance system of a healthcare unit, EPPs are useful for identifying and correcting practice deviations at an early stage. They should be regularly repeated and combined with rigorous training and qualification of operators involved in RPs injection.

## 1. Introduction

As a specific activity in hospital pharmacy, radiopharmacy essentially consists of the management of the radiopharmaceuticals (RPs) circuit [1]. Due to the unique nature of this activity and regarding the constraints related to the handling of unsealed radioactive sources for both diagnostics and therapy [2], radiopharmacy is mainly practiced within nuclear medicine (NM) departments, directly in the care unit where RPs are administered to patients. The radiopharmacist, a specialized hospital pharmacist who guarantees the quality and safety of the radiopharmaceutical drugs process, therefore, has significant potential for clinical pharmacy and interdisciplinary missions within the NM department [3,4]. In conventional NM, most RPs are preparations, produced on a daily basis through the radiolabeling of a vector molecule (e.g., a bisphosphonate in the case of bone scintigraphy) by a gamma-emitting radioelement (e.g., metastable technetium-99). These preparations, conditioned in multidose vials, are then dispensed in patient-name syringes, delivered as needed, and administered by injection. As such, the administration of RPs is a key step in both the radiopharmaceutical drug circuit and the management of patient care in NM. Indeed, any error occurring during this step and any previous deviation not identified at this step may result in a medication error, potentially causing harm to the patient. The most common errors encountered during the radiopharmaceutical injection phase are closely related to the five rights of medication administration [5]. They may involve patient identity mistakes, failure to check the RP and/or the radioactive dose in the syringe at the time of injection [6], lack of asepsis during injection [7], or lack of radiation protection that may overexpose the patient and/or the operator [8]. It is therefore important to ensure the safety of the RPs injection step and to maximize the quality of patient care through the training and qualification of the healthcare staff (in this case, nuclear medicine technologists (NMTs)).

Healthcare audits are practice evaluation methods based on specific criteria that measure the differences between the actual practice observed and the expected or recommended practice. Among these methods, the evaluation of professional practices (EPP) consists of analyzing a clinical activity in relation to available and updated professional recommendations, to set up an improvement action plan to optimize this professional activity and the quality of care delivered to patients. In a quality management approach, several methodologies for the evaluation of professional practices in radiopharmacy were reported in the literature; however, these did not include the RPs administration step [9,10,11]. Thus, we describe herein the development and implementation of an audit program focused on the injection step of RPs for scintigraphy. This audit was registered within our center as a program of institutional EPP and aimed at identifying possible non-conformities to good practices, for which actions for improvement could be proposed.

## 2. Materials and Methods

### 2.1. Elaboration of the Evaluation Grid

Firstly, the scope of the audit in development was defined on the basis of several guidelines in NM and radiopharmacy [8,12,13]. Then, the evaluation grid to be used during the audit (see Table S1) was elaborated by a working group composed of a quality officer, two radiopharmacists, a health executive NMT, a nuclear physician, and a medical physicist.

To ease the audit process, the chart items were divided into 4 chronological steps: patient reception, administration phase, post-administration phase, and theoretical radiation protection questioning. Each item on the chart was also assigned to one of the four main themes evaluated during the audit: reception and administration, identity vigilance, radiation protection, or hygiene. Finally, each item on the grid was weighted by a coefficient ranging from 1 to 3 depending on its criticality (1 being the least critical and 3 the most critical), as subjectively assessed by the working group. Several items were outside both the scope of the themes evaluated and the scoring system, their purpose being to describe the injection conditions or the patient population being managed (e.g., injection site or level of disability of patients).

From a practical point of view, this audit campaign was planned over a period of one month to allow for the evaluation of all NMTs who might be required to inject RPs into our unit. The evaluation included observation of the NMT during 4 RPs injections: usually 4 intravenous (IV) injections for scintigraphy purposes, or 3 IV injections for scintigraphy and 1 subcutaneous injection for lymphoscintigraphy purposes, when possible. A final interview with the NMT completed the evaluation. The following rule was applied to score the criteria of the audit grid:

- Criterion met (O) if all aspects of the item were fulfilled (the item scores the totality of the points related to its weighting);
- Criterion partially met (I) when one of the aspects of the item was not satisfactory (the item scores half of the points related to its weighting);
- Criterion not met (N) when none of the aspects of the item were correctly covered (the item scores no points);
- Not applicable criteria (NA) when the aspects related to the item are not relevant to the activity audited; NA criteria are therefore not considered in the calculation of rating scores.

To facilitate the calculation of rating scores and the interpretation of the results obtained within the audited group of NMTs, a computer-based spreadsheet was created, allowing the recording and archiving of the results collected for 10 NMTs during each audit cycle (see Table S2). The total score in percentage, calculated for each injection audited and considering the weighting of each item, was presented to the operator at the end of the 4 evaluations. An individual score for each of the 4 themes audited, calculated in the same way, was also provided.

### 2.2. Audit Process

The evaluation was conducted in the NM department of a cancer center, which performs about 6000 general NM imaging and 5000 positron emission tomography (PET) imaging annually. The NM unit had 10 NMTs, among which 9 performed RPs administration. These 9 NMTs had between 2 and 26 years of experience (mean = 12.9 ± 9.3 years) in NM practice. For each audit, the examiner team was composed of the same two radiopharmacists. The evaluation of 4 administrations and the interview with the audited NMT were organized over a morning period and lasted 2 h on average. An audit grid was completed by each radiopharmacist while observing the events listed among the items, as they occurred. In case of a discrepancy between the two auditors in scoring a criterion of the audit grid, immediate discussion to resolve the disagreement took place as soon as the audited injection was finished. At the end of the whole audit campaign, the overall results obtained by each NMT were presented anonymously during a staff meeting, with the listing of the partially compliant and non-compliant criteria encountered. In response, to improve the relevance of the corrective actions that could be proposed, each NMT was asked to comment on the potential risk of an undesirable event associated with the non-conformities encountered and on the possible improvements that they would like to be implemented.

### 2.3. Data Analysis

The data obtained during the audit campaign were recorded in the computer-based spreadsheet created for this purpose (see Table S2). For each evaluation, the number and proportion of criteria scored “O”, “I”, “N” and “NA” was expressed in absolute value and percentage, respectively. A total score, expressed in percentage, was then calculated considering the weight and the score of each criterion. An average score per operator, expressed in percentage ± standard deviation, was finally calculated from the results of four injection audits. The overall results and the NMT’s responses were compiled and reviewed by the working group to provide improvement suggestions for each non-compliant or partially compliant criterion.

## 3. Results

The working group collaboratively developed a 36-item grid, consisting of 13 items for the Patient reception step, 15 items for the Administration step, 3 items for the post-administration step, and 5 items for the Theoretical radiation protection questioning (see Table S1). Concerning the 4 themes considered, the subdivision of some items into sub-idem increased the number of scored criteria to 51, respectively 7 criteria related to Reception and administration, 9 criteria related to identity vigilance, 15 criteria related to radiation protection, and 20 criteria related to hygiene. Almost half of these criteria (n = 25) were weighted with an intermediate criticality (weight = 2), 11 were considered highly critical (weight = 3) and 15 qualified as low critical (weight = 1). Seventeen criteria were not related to any of the themes studied, 7 to characterize the patient population under care, and 10 to specify the conditions of administration. Table 1 summarizes the structure of the audit grid.

**Figure 1:**
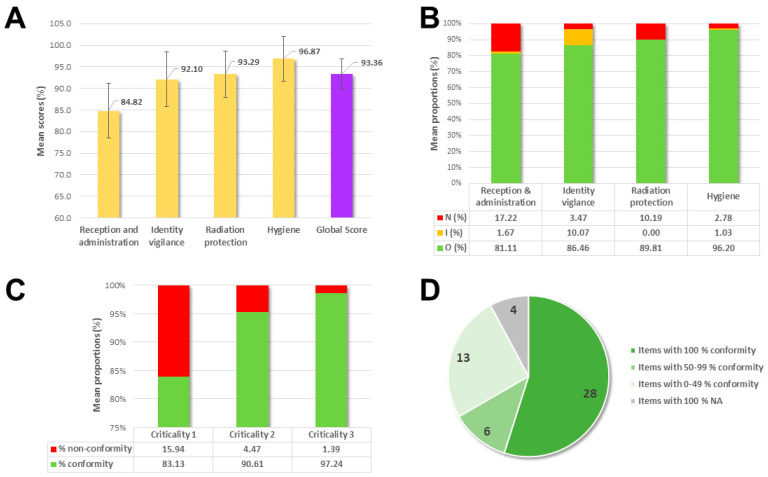
Results of the 36 RP injections audited during the EPP campaign with (**A**) Mean scores (%) in total and according to the themes evaluated; (**B**) Mean proportions (%) of unmet, partially met and compliant criteria based on the theme assessed; (**C**) Mean proportions (%) of conform (compliant) and non-conform (unmet or partially met) criteria depending on their criticality; (**D**) Distribution (n) of items according to their conformity rate.

The entire NMT team of our unit (n = 9) was audited over 1 month. The detailed results obtained are summarized in Figure 1. Of the 36 injections evaluated, 33 (91.67%) were conventional intravenous injections for scintigraphy and only 3 (8.33%) were subcutaneous injections for lymphoscintigraphy. The average total score, on all themes evaluated, for the 36 injections observed was 93.36% ± 3.52% (min-max: 85.87–97.20%) (Figure 1A). The average proportion of unmet and partially met items during one injection was 7.01% (min-max: 0–20.51%) and 3.00% (min–max: 0–8.82%), respectively. The themes for which the proportions of unmet items were the most important were Reception and administration (17.22%) and Radiation protection (10.19%) (Figure 1B). The proportion of conformity of the studied items seemed to be related to their criticality, with 97.24% of the criteria of high criticality in conformity, 90.61% of the criteria of intermediate criticality in conformity, and 83.13% of the criteria of low criticality in conformity (for 1.39%, 4.47% and 15.94% of non-conformity, respectively) (Figure 1C). Out of the 4 themes evaluated, 12 criteria (23.53%) had a non-conformity rate above 50%, including the introduction of the NMT (83.3% non-conformity, n = 29/36), the use of a lead apron (55.6%, n = 20/36), the questioning of patients of childbearing age about potential breastfeeding (83.3%, n = 5/6) or the proper 4-step detersion when placing a catheter (100%, n = 5). Conversely, 28 of the criteria assessed (54.90%) had a compliance rate of 100%, 6 criteria (11.76%) had a compliance rate between 50% and 99%, and 13 criteria (25.49%) had a compliance rate between 0% and 49% (Figure 1D).

Regarding the patient population managed, the majority (n = 34/36, 94.4%) were valid patients; two patients in a wheelchair (2.8%) were also managed. Only one patient (2.8%) verbally expressed anxiety about the examination. Most patients were administered in a conventional injection booth (n = 33/36, 91.7%), 2 patients (5.6%) were managed in an adjoining injection box normally used for therapies administration, and 1 patient (2.8%) was injected directly under the camera. During the injections audited, none of the deviations identified had a direct impact on the appropriate management of the patient within the NM unit.

During the staff meeting to report the results of this EPP, all the evaluated criteria were reviewed, with a particular emphasis on those listed above with the highest non-conformity rates. These partially compliant and non-compliant criteria are summarized in Table 2. The main points of vigilance that emerged were related to identity vigilance items (items numbers 2, 5, 8, 24, and 25) and hygiene items (items numbers 15, 16, and 21).

In response to the suggestions and requests of the NMTs, several measures for improvement emerged to address the deviations encountered. Theoretical reviews focused on hygiene and radiation protection were given in small groups of 3 NMTs by the radiopharmacists and the medical physicist of the NM unit, respectively; these interventions have been instituted with an annual frequency. Moreover, a poster reminding the five rights of medication administration was designed and placed in each injection room of our unit (see Figure S1).

The results of this monocentric study provide a snapshot of how NMTs manage patients in our unit when injecting an RP drug. Some deviations may not be identified by this audit, especially since we describe here its first year of implementation. In addition, since the audit grid was designed according to our internal procedures, it may require a slight revision before being transposed to another center. Concerning the results presented above, they may be slightly distorted by the Hawthorne effect, a reference to the propensity of individuals to behave more appropriately when they know they are being observed [14]. Lastly, this type of evaluation tends to essentially highlight major deviations and can hardly identify minor practice deviations, although their rapid correction after detection would ensure the best possible quality of patient care.

## 4. Discussion

In view of the very specific activities carried out in NM departments, ensuring the safety of both the patient care process and the RPs circuit is a major and daily concern in this discipline [15]. Within this context, Kasalak et al. examined in a recent study the patient safety incidents recorded in the incident reporting system of their nuclear medicine unit [16]. Medication was identified as the main type of incident (24.5%), followed by clinical administration errors (19.0%) and clinical procedure issues (18.4%). Several security measures can therefore be considered to ensure the safety of patient management in NM, particularly at the RPs administration step. A commonly accepted action might be, for example, the implementation of an efficient quality management system [17]. It should also imply strict adherence to validated internal procedures and extensive operator training, which can sometimes be lacking [18]. The creation of electronic connections between management systems (e.g., integrative NM/radiopharmacy software or bar code system for RPs tracking) can also play an important role in securing both the medication circuit and the patient course [19,20]. Nevertheless, the risk of administration errors in NM can never be totally excluded, as their causes are multiple, most often involving either the radiopharmaceutical used (incorrect RP or activity) or the patient being managed (incorrect patient injected) [21]. Kearney and Denham published a study of the NM errors reported in Australian radiation incident registers in 2016 [22]. They highlighted that the primary cause of radiation incidents in NM was a failure in patient management before or during the administration of the RPs. Comparable results have already been shown by Martin, who studied an incident reporting system in Scotland and evidenced that 47% of radiation incidents in NM were due to the administration of the incorrect radiopharmaceutical or incorrect dose [23]. Previous studies by Larcos et al. rather tended to show that the preparation and dispensing of RPs was the riskiest step in the process (n = 71/149 maladministrations), however, injection errors still accounted for 26.8% of the maladministration reports studied (either injection of the wrong RP, n = 27/149, or injection to an incorrect patient, n = 13/149) [24,25]. This underlines the importance of securing the RP administration step, specifically the patient reception and pre-injection phase. More generally, this securing approach should also be extended to other NM activities such as targeted radionuclide therapy (TRT), where an error would be highly detrimental to the patient, and even more so considering the growing interest for new intravenous TRT drugs (e.g., ^177^Lu-DOTATATE, ^177^Lu-PSMA-617 or even alpha emitters such as radium-223 dichloride or ^225^Ac-DOTATATE) [26].

Given the lack of RPs administration audit methodology in the literature, we designed an easy-to-use tool to identify deviations in RPs administration practices. Within an NM unit, the radiopharmacist appears particularly qualified to conduct this type of quality assurance and proper use of medicinal products mission. An evaluation led by a team of two radiopharmacists also limits subjectivity bias. The audit grid was developed by selecting specific key criteria to maintain a relevant evaluation, while reducing the time-consuming aspect of such audits. Considering the variability of care procedures between centers and the diversity of NM activities, this grid can be modulated or even adapted to other NM tasks such as PET RPs injection.

With an average score per operator above 90%, the results of this first audit campaign within our NM unit are encouraging. Interestingly, global scores were not correlated with the years of experience of the NMTs (R² = 0.1); nevertheless, several deviations from good practice were highlighted. About the reception of the patient and identity vigilance concerns, it appeared necessary to reconsider the nature of the initial interactions with the patient in order, firstly, to limit as much as possible the risks associated with identity vigilance and, secondly, to better promote patient-centered care in NM, proven to have a positive impact in patient management [27]. Just prior to administering an RP drug, increased vigilance was requested for the final check between the examination, the RP syringe label, and the patient’s identity. Indeed, even if the overall non-compliance of this item (n°25) was only 11.1%, it describes the last time point before injection where potential discrepancies can be identified and corrected.

The improvement action plan set up in accordance with the results of this audit included hygiene (with a particular emphasis on the proper disinfection associated with peripheral IV catheter insertion) and radiation protection reminders, whose benefits will have to be evaluated on a longer term with annual repetitions of this EPP. These theoretical and procedural reminders also called for the promotion of flexibility and resilience within the NMTs team, in an attempt to optimize the security of the RPs injection position [28].

## 5. Conclusions

This work led to the development of an audit tool for EPP at the administration step of injectable RP drugs. Implementation of this method in the NM department of a cancer center revealed several non-conformities, notably on admission and injection, patients’ identity monitoring, and radiation protection points. These results led to the set-up of an improvement action plan with corrective measures to address the identified gaps. The annual repetition of this EPP on RPs injection practices would be especially useful for the early identification and correction of practice discrepancies; moreover, future EPP campaigns using this tool could be extended to more than one center with the possibility of cross-evaluation. As a complement to other global audit approaches in nuclear medicine and radiopharmacy [29,30], this work provides a turnkey EPP methodology focused on a critical step of patient care in NM. It addresses the constant need for evaluation and requalification of paramedical workers, in a positive error culture, to avoid deviations in healthcare practices. This tool can be part of the continuous quality improvement process of a hospital care unit, as defined by several certification authorities, and can be a support for departments willing to undertake such processes.

## Figures and Tables

**Table 1:** Distribution of the audited items according to their theme and criticality.

	Topics	Reception and Administration		Identity Vigilance		Hygiene		Radiation Protection		Total	
Criteria and Criticality											
Total number of criteria in the category	High criticality (3)	7	0	9	2	20	5	15	4	**51**	**11**
	Intermediate criticality (2)		1		7		10		7		**25**
	Low criticality (1)		6		0		5		4		**15**

**Table 2:** Details of the most critical non-compliant criteria identified during the EPP campaign.

Theme	Item	Partially Compliant Records (n)	Non-Compliant Records (n)	Overall Non-Compliance Proportion (%)
A	The nuclear medicine technologist introduces himself	1/36	29/36	83.3
	In case of injection of blood-derived drugs: verbal information of the patient before injection	0	2/3	66.7
	In case of injection of blood-derived drugs: written information provided to the patient	0	2/3	66.7
I	Concordance with the examination checked in the patient file	0	5/36	13.9
	Once installed, the patient asked to confirm their last name	0	3/36	8.3
	Once installed, the patient asked to confirm their first name	0	2/36	5.6
	Patient was asked to confirm the purpose of the examination by open question (O)/By closed question (I)	25/36	0	69.4
	Concordance between the examination and the radiopharmaceutical checked just before administration	4/36	0	11.1
R	Woman of childbearing age asked about breastfeeding	0	5/6	83.3
	Secure transport of the RP syringe	0	1/1	100
	Shielded case used for RP syringe transport	0	1/1	100
	Mobile cart used for RP syringe transport	0	1/1	100
	Shielded apron worn for RP injection	0	20/36	55.6
H	Preparation of an injection tray with pads, plasters, needles, gloves, tourniquet, and syringe	5/36	0	13.9
	Catheter placed after 4-step detersion	0	5/5	100
	Check of the catheter line by opening infusion of NaCl 0.9%	0	4/5	80

A = Reception and administration; I = Identity vigilance; R = Radiation protection; H = Hygiene.

## Data Availability

Data supporting reported results are available within the article or as supplementary data.

## Supplementary Materials

The following supporting information can be downloaded at: https://www.mdpi.com/article/10.3390/healthcare10112247/s1, Table S1: EPP evaluation grid; Table S2: computer-based spreadsheet for EPP results recording; Figure S1: five rights of medication administration poster.

## References

[1] Bhonsle J., Chianelli M., Hartman N.G., Jeong J.M., Ozker K., Savio E., Saw M.M., Solanki K.K. (2008). Operational Guidance on Hospital Radiopharmacy: A Safe and Effective Approach.

[2] Shukla U., Chowdhury I.H., Beckta J.M., Witt J.S., McFarlane M., Miller C.J., Huber K.E., Katz M.S., Royce T.J., Chowdhary M. (2021). Unsealed source: Scope of practice for radiopharmaceuticals among United States radiation oncologists. Adv. Radiat. Oncol..

[3] Dinet J., Becker S., Le Cloirec J., Bohn P. (2020). The added value of clinical radiopharmacists in Nuclear Medicine: The example of glomerular filtration rate assessment in kidney donors. J. Clin. Pharm. Ther..

[4] Leclerc P., Marie S., Fouque J., Olivier M., Blondeel-Gomes S. (2021). How can we optimise the pharmaceutical analysis of radiopharmaceutical pediatric prescriptions?. Eur. J. Hosp. Pharm..

[5] Kim J., Bates D.W. (2013). Medication administration errors by nurses: Adherence to guidelines. J. Clin. Nurs..

[6] Williams E.D., Harding L.K. (1995). Radiopharmaceutical maladministration: What action is required?. Nucl. Med. Commun..

[7] Joint Working Party of the NSW Branch of ANZSNM and HURSOG (1999). Proposed guidelines for the administration of diagnostic therapeutic radiopharmaceuticals. ANZ Nucl. Med..

[8] Marengo M., Martin C.J., Rubow S., Sera T., Amador Z., Torres L. (2022). Radiation safety and accidental radiation exposures in nuclear medicine. Semin. Nucl. Med..

[9] Potdevin-Verdier J., Le Meur C., Maget A., Calas-Chane-Woon-Ming L., Carre A.-L., Galvez D., Hammer-Lefevre C., Lao S., Debordeaux F., Véran N. (2021). Validation of a professional practice assessment tool in radiopharmacy and results of a multisite study. Pharm. Hosp. Clin..

[10] Ben Reguiga M., Rotaru I., Ait Abdeslam S., Pons-Kerjean N. (2016). EPP en radiopharmacie: Expérimentation d’un outil international d’auto-évaluation des pratiques. Pharm. Hosp. Clin..

[11] Ebel-Lao S., Collomp R., Dompe J., Ruitort S., Carrier P., Girma A., Koulibaly P., Darcourt J., Mousnier A. (2008). Formation initiale et continue des préparateurs en radiopharmacie: Mise en place d’une démarche qualité. J. Pharm. Clin..

[12] World Health Organization (2010). WHO Best Practices for Injections and Related Procedures Toolkit. No. WHO/EHT/10.02.

[13] Gillings N., Hjelstuen O., Ballinger J., Behe M., Decristoforo C., Elsinga P., Ferrari V., Peitl P.K., Koziorowski J., Laverman P. (2021). Guideline on current good radiopharmacy practice (CGRPP) for the small-scale preparation of radiopharmaceuticals. EJNMMI Radiopharm. Chem..

[14] McCambridge J., Witton J., Elbourne D.R. (2014). Systematic review of the Hawthorne effect: New concepts are needed to study research participation effects. J. Clin. Epidemiol..

[15] Giannoula E., Panagiotidis E., Katsikavelas I., Chatzipavlidou V., Sachpekidis C., Bamidis P., Raftopoulos V., Iakovou I. (2020). Quality & safety aspects of nuclear medicine practice: Definitions and review of the current literature. Hell. J. Nucl. Med..

[16] Kasalak Ö., Yakar D., Dierckx R.A.J.O., Kwee T.C. (2020). Patient safety in nuclear medicine: Identification of key strategic areas for vigilance and improvement. Nucl. Med. Commun..

[17] Dondi M., Paez D., Torres L., Marengo M., Delaloye A.B., Solanki K., Van Zyl Ellmann A., Lobato E.E., Miller R.N., Giammarile F. (2018). Implementation of quality systems in nuclear medicine: Why it matters. An outcome analysis (quality management audits in nuclear medicine Part III). Semin. Nucl. Med..

[18] Larcos G., Prgomet M., Georgiou A., Westbrook J. (2017). A work observation study of nuclear medicine technologists: Interruptions, resilience and implications for patient safety. BMJ Qual. Saf..

[19] De Neef L., Peyronnet D., Blondeel-Gomes S. (2020). How to validate radiopharmaceuticals management software?. Pharm. Technol..

[20] Hakala J.L., Hung J.C., Mosman E.A. (2012). Minimizing Human Error in Radiopharmaceutical Preparation and Administration via a Bar Code-Enhanced Nuclear Pharmacy Management System. J. Nucl. Med. Technol..

[21] Yenson T., Larcos G., Collins L.T. (2005). Radiopharmaceutical maladministrations in New South Wales. Nucl. Med. Commun..

[22] Kearney N., Denham G. (2016). Recommendations for nuclear medicine technologists drawn from an analysis of errors reported in Australian radiation incident registers. J. Nucl. Med. Technol..

[23] Martin C.J. (2005). A survey of incidents in radiology and nuclear medicine in the west of Scotland. Br. J. Radiol..

[24] Larcos G.S., Collins L.T., Georgiou A., Westbrook J.I. (2014). Maladministrations in nuclear medicine: Revelations from the Australian radiation incident register. Med. J. Aust..

[25] Larcos G., Collins L.T., Georgiou A., Westbrook J.I. (2015). Nuclear medicine incident reporting in Australia: Control charts and notification rates inform quality improvement. Intern. Med. J..

[26] Sgouros G., Bodei L., McDevitt M.R., Nedrow J.R. (2020). Radiopharmaceutical therapy in cancer: Clinical advances and challenges. Nat. Rev. Drug. Discov..

[27] Berman J., Moadel R.M., Goldman-Yassen A.E., Abraham T., Ye K., Volansky J., Goldberg-Stein S. (2020). Impact of patient-centered care on the patient experience in nuclear medicine. Curr. Probl. Diagn. Radiol..

[28] Braithwaite J., Wears R.L., Hollnagel E. (2015). Resilient health care: Turning patient safety on its head. Int. J. Qual. Health Care.

[29] Dondi M., Torres L., Marengo M., Massardo T., Mishani E., Van Zyl Ellmann A., Solanki K., Bischof Delaloye A., Lobato E.E., Miller R.N. (2017). Comprehensive auditing in nuclear medicine through the international atomic energy agency quality management audits in nuclear medicine (QUANUM) program. Part 1: The QUANUM program and methodology. Semin. Nucl. Med..

[30] Gillings N., Hjelstuen O., Behe M., Decristoforo C., Elsinga P.H., Ferrari V., Kiss O.C., Kolenc P., Koziorowski J., Laverman P. (2022). EANM guideline on quality risk management for radiopharmaceuticals. Eur. J. Nucl. Med. Mol. Imaging.

