# Supplementary material for: Development and Implementation of a Professional Practices Evaluation during Radiopharmaceuticals Administration
Source: Healthcare (Basel). 2022 Nov 10;10(11):2247. doi: 10.3390/healthcare10112247 (PMC9690994; doi:10.3390/healthcare10112247)
Supplement: Supplementary file 1 [file healthcare-10-02247-s001.zip › Suppl data Figure S1.pptx]

## Slide 1
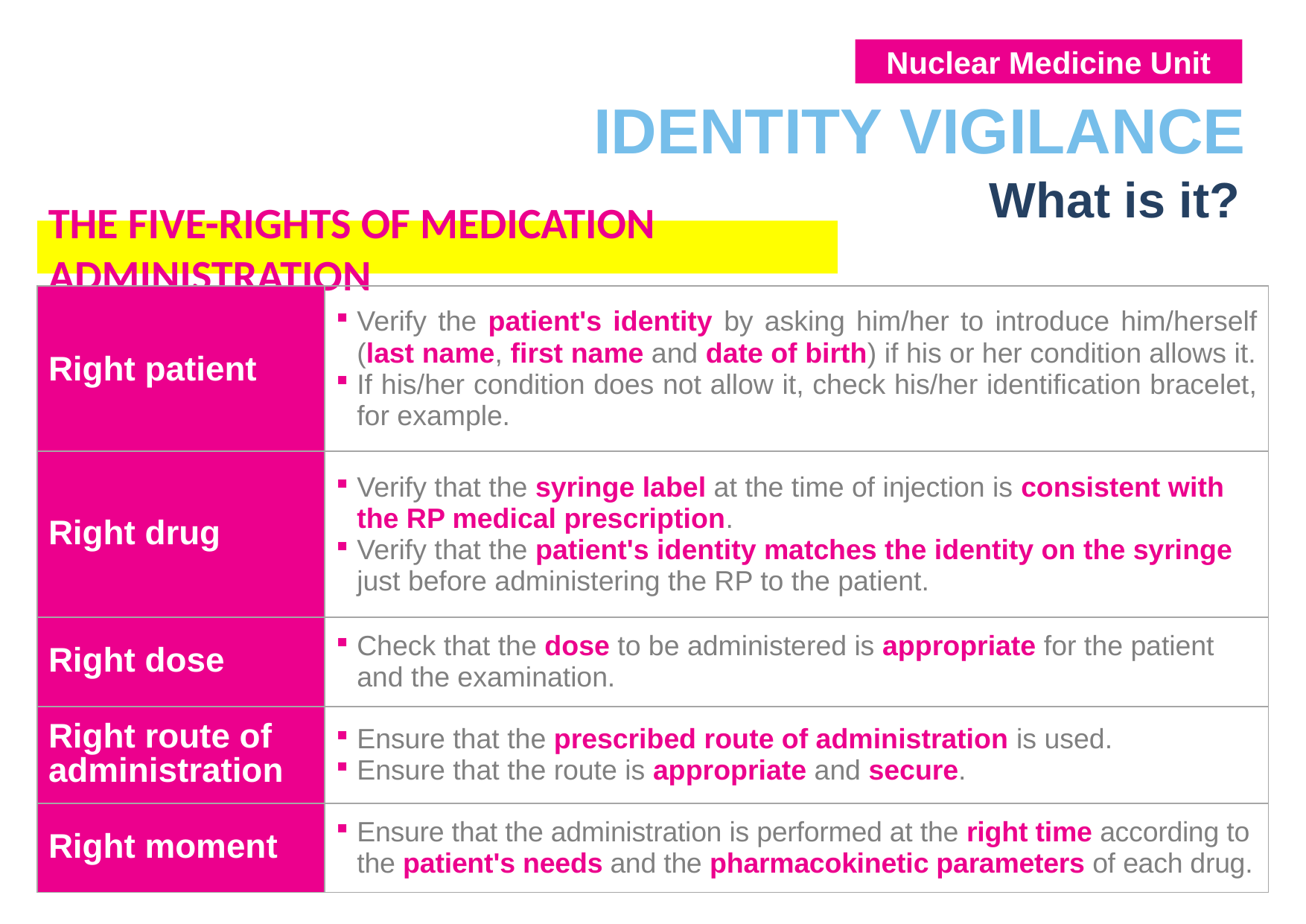

Nuclear Medicine Unit
IDENTITY VIGILANCE
What is it?
The five-rights of medication administration
| Right patient | Verify the patient's identity by asking him/her to introduce him/herself (last name, first name and date of birth) if his or her condition allows it. If his/her condition does not allow it, check his/her identification bracelet, for example. |
| --- | --- |
| Right drug | Verify that the syringe label at the time of injection is consistent with the RP medical prescription. Verify that the patient's identity matches the identity on the syringe just before administering the RP to the patient. |
| Right dose | Check that the dose to be administered is appropriate for the patient and the examination. |
| Right route of administration | Ensure that the prescribed route of administration is used. Ensure that the route is appropriate and secure. |
| Right moment | Ensure that the administration is performed at the right time according to the patient's needs and the pharmacokinetic parameters of each drug. |
